# Cytogenetic impact of gamma radiation and its effects on growth, yield and drought tolerance of maize (*Zea mays* L.)

**DOI:** 10.1186/s12870-025-06111-x

**Published:** 2025-02-03

**Authors:** Walaa M. Al-Sayed, Hanaa H. El-Shazly, Awatif I. El-Nahas, Ahmed A. A. Omran

**Affiliations:** Department of Biological and Geological Sciences, Faculty of Education, Ain Shams University, Cairo, Egypt

**Keywords:** Maize, Gamma radiation, Drought, Growth and yield traits, Chromosomal aberrations, RT-PCR, SDS-PAGE

## Abstract

Maize is the third most important grain crop worldwide after wheat and rice; it is a vital global crop, serving as a key source of food, animal feed, and industrial products, making it essential for food security and economic stability in many countries. Drought stress adversely affects water uptake and can stunt growth, reducing the overall productivity of maize. So, this study was carried out to investigate the cytogenetic effects of gamma radiation and drought stress on maize SC131 genotype, focusing on chromosomal aberrations in seedling root meristems induced by varying doses of gamma irradiation (50, 100, 150, 200, and 250 Gray) and drought stress imposed by 10% polyethylene glycol (PEG). The present study also aims to evaluate the impact of these treatments on growth parameters under a controlled pot experiment. Additionally, molecular polymorphism induced by both gamma irradiation and drought stress was analyzed using Real-Time quantitative PCR techniques for *DREB2*, *ERF*, and *EF* transcription factors. Also, under a field condition experiment, maize plants were subjected to the same gamma irradiation doses and drought stress by reducing the number of irrigations, with subsequent evaluations of yield attributes to assess the overall impact of treatments on plant performance. The study also investigates the sodium dodecyl sulfate–polyacrylamide gel electrophoresis (SDS-PAGE) banding patterns of proteins in grains yielded under the influence of gamma radiation and drought treatments. Findings of the current investigation indicate that the low dose of gamma radiation (50 Gray) not only induces cytogenetic changes but also enhances drought tolerance and improves yield characteristics, suggesting that targeted gamma irradiation could serve as a viable strategy to bolster maize resilience in challenging environmental conditions.

## Introduction

Maize (*Zea mays* L.) is a diploid (2n = 2x = 20), annual, monoecious cereal of the family Poaceae (Gramineae). Maize is the third most important grain crop worldwide after wheat and rice [1]. It is widely farmed over the world in a variety of agro-ecological conditions. Maize, a C4 species, uses moisture and sunlight efficiently to produce high yield and total dry matter [2]. Global maize production is becoming increasingly important as a source of food, forage, oil, and biofuel for the world's growing population. Where, every year, around 1,137 million tons of maize are produced over the world [3].

Drought harms the growth and reproduction of maize, because a water deficit can greatly cause the following reduction in maize: seed germination, seedling growth, leaf expansion, shoot and root growth, early maturation, plant height, photosynthesis, nutrient uptake, pollination, fertilization, and grain yield; thus proper water management is critical to maize growth [4, 5]. Furthermore, drought stress can decrease the initiation of flowering in male flowers and generate variables that interfere with pollen formation [6, 7]. It is therefore important to increase the productivity of maize, while maintaining them in the face of persistent drought caused by water scarcity and changing the climate. That requires a strategy to develop drought-tolerant maize plant that can effectively respond to water shortages and climate change [8].

Gamma rays are electromagnetic radiation, commonly used as mutagens due to their ease of application, good penetration, reproducibility, high mutation frequency, and fewer disposal problems [9]. Gamma rays' biological effect is dependent on their interaction with atoms or molecules in the cell, notably water, which produces free radicals [10]. These radicals can harm or disrupt essential components of plant cells and have been documented to influence the morphology, anatomy, biochemistry, and physiology of plants in different ways depending on the radiation dose [11]. These impacts include changes in the plants' cellular structure and metabolism, such as dilatation of thylakoid membranes, variation in photosynthesis, modulation of the antioxidant system, and accumulation of phenolic chemicals [12]. Low-dose ionizing irradiation on plants manifests as accelerated cell proliferation, germination rate, cell growth, enzyme activity, stress resistance, and crop yields [13]. Gamma irradiation is more effective for improving the qualities and self-life of grains and fruits and can increase genetic diversity for traditional breeding to scrutinize new genotypes with better traits, such as stress tolerance, grain yield, and quality [9]. In addition, gamma rays are essential physical activators for crop plant growth, yield attributes, and yields without using external fertilizers. The use of low doses of gamma irradiation in combination with drought stress displays better performance than that of the non-irradiated plants under the same condition [14].

A cytogenetic analysis is important in assessing genetic impact of the chemical and physical mutagens [15]. These changes provide the basis for introducing genetic variability in many plant traits [16]. The most common influence of γ-irradiation is disturbing mitotic activity inducing chromosomal aberration, and affecting yield. The most common chromosomal changes recorded in response to γ-irradiation is the production of mitotic chromosomal aberrations; stickiness, lagging chromosomes and bridges at anaphase and telophase [17]. However, the only recoverable chromosomal rearrangements are those that can replicate DNA molecules and hence can be stably passed on to the next generation [18].

Sodium dodecyl sulfate polyacrylamide gel electrophoresis (SDS-PAGE) of storage seed protein was used to differentiate between cultivars of *Zea mays* and to identify inbred lines [19]. Protein electrophoresis is a practical biochemical technique and reliable method to detect biochemical markers in many crops. It has been used to provide a relatively convenient and rapid method of identification and classification of gene bank collections and to distinguish between maize genotypes [20, 21]. On the other hand, electrophoresis techniques provide a unique method to study protein substructure differences among genotypes and treatments, enabling the study of hybrids and lines [22].

Gene expression analysis is one of the most significant methods for understanding the complex signaling networks that control the many responses observed during the plant life cycle or when exposed to diverse stimuli [23]. Real-Time quantitative PCR (RT-qPCR) stands out from other gene expression methodologies due to its accuracy, sensitivity, and speed. As a result, the method has emerged as the gold standard for medium-throughput gene expression analysis [24]. Transcription factors emerged as critical regulators in diverse signaling networks, playing a crucial role in enhancing plant growth and development under stress situations [25]. Major plant transcription factor families, such as, *DREB1*/*CBF*, *DREB2*, *AREB*/*ABF*, *NAC*, *AP2*/*ERF*, and *MYB*, have been documented as key regulators in plant responses to various abiotic stresses [26]. RT-qPCR was utilized to confirm the expression levels of target genes. To minimize biased results during RT-qPCR analysis, a normalization step of the gene expression data is required to compensate for differences between samples and conditions. Normalization during RT-qPCR analysis is typically accomplished using a reference gene (Housekeeping gene) that must be expressed at constant levels regardless of experimental settings, cell types, tissue, developmental stage, or stress treatment [27].

The current study evaluates the effects of irradiation accompanied by drought stress treatments on maize, to test the impacts of low doses of gamma radiation, on the germination and growth parameters of SC131 maize genotype under control and drought conditions. In this respect, the current study aimed at, (1) study the cytogenetic effects, i.e., chromosomal aberrations of the gamma irradiation doses and treatments of drought induced by 10% PEG, in the seedling root meristems of maize genotype, (2) evaluate the impact of the same doses and drought treatments on germination and early seedling growth parameters in a pot experiment, (3) estimate molecular polymorphism induced by both gamma irradiation and drought treatments using *DREB2*, *ERF* and *EF* genes as molecular markers by Real-Time quantitative PCR techniques, (4) examine the impact of both gamma irradiation and drought on yield components and its attributes of field-grown plants, (5) Study the SDS-PAGE banding pattern of yielded grains protein under the effect of gamma irradiation and drought treatments.

## Materials and methods

### Plant material and gamma irradiation

Egyptian single cross of maize SC131 (Pedigree: Sakha9 x Sakha5) was used in this study. Grains of this genotype was obtained from the Maize Research Department, Field Crops Research Institute of the Agricultural Research Center (ARC) in Giza, Egypt. This genotype was selected from the germplasm based on its agronomic importance as well as its disease-resistant and drought-tolerant properties. Grains were exposed to five doses of gamma irradiation using the Indian Gamma Cell Unit (IGCU) at the Atomic Energy Commission (AEC), Nasr City, Cairo, Egypt, using Co60 as a source of irradiation. The applied doses were 50, 100, 150, 200, and 250 Gray (Dose Rate = 0.960 KGy/h). Grains of control samples were not exposed to gamma irradiation.

### Cytological procedures

For the cytological analyses, two groups were used, first group comprised maize grains that were treated with gamma irradiation doses only and the second group that was treated with the same doses of radiation accompanied by the exposure to simulated drought using PEG-6000 (10%). Seeds were germinated on filter paper in Petri dishes, control groups were moistened with distilled water, and both groups were grown as factorial experiment under completely randomized design with three replications at ± 25 °C. Washed root tips of 1–2 cm, from each treatment were fixed in a 3:1 (v/v) ethanol/glacial acetic acid mixture for 24 h and finally stored at 4°C in a 70% ethanol solution. Fixed root tips were hydrolyzed in 1N HCl at 60°C for 10 min right before examination, and the hydrolysis solution was then replaced by Feulgen stain for around 3–4 h as described in Darlington and La Cour [28]. After being cut and squashed between a slide and a cover in a drop of 45% acetic acid, the strongly stained meristematic region was examined under a light microscope at a magnification of 1000 times. A total of 5000 cells from both the control and treatment concentrations were scored. By dividing the number of dividing cells by the total number of cells tested, the mitotic index (M.I.) was computed as a percentage, as well as the percentages of mitotic phases. In each stage, abnormalities were counted and expressed as a proportion of dividing cells.

### Simulated drought experiment and plant growth attributes

Grains were planted in 10 pots replica, each pot (25 cm in diameter and 25 cm in depth) containing 3.5 kg of homogeneous loamy clay soil. Ten grains were sown in each pot. Pots were divided into two groups of treatments. The first treatments were exposed to seven doses of gamma irradiation and irrigated by distilled water, and the second treatments was exposed to the same doses of radiation accompanied with simulated drought stress imposed by 10% of polyethylene glycol (PEG). Grains of control were not exposed to any treatment. After 21 days of planting, morphological parameters were evaluated for 10 plants randomly selected for each treatment. These parameters are root length, root fresh weight and root dry weight, shoot length, shoot diameter, fresh and dry weight of shoot, number of leaves and leaf surface area. Morphological parameters were assessed according to the International Seed Testing Association (ISTA) rules [29].

### Real‑time PCR reactions

Total RNA was extracted using TRIzol reagent (Ambion, USA), and first-strand cDNA synthesis was carried out using 5 mg of total RNA and a 20-bp poly (dT) oligonucleotide, following the Reverse Transcription System procedure (Promega, USA). The SYBR green method was used for quantitative RT-PCR analysis on the CWBIO Fast SYBR Mixture real-time PCR system (CWBIO, China). The primers used in this study are listed in Table 1. Cycle Threshold (CT) was calculated based on the default setting of real-time software sequence detection results. The gene expression in the form of relative quantification was estimated using Ct values. The fold change of target genes compared to the control was calculated according to the Livak method 2^−∆∆Ct^ [30]; where:

$$\Delta\mathrm{CT}\:=\:\text{CT}\;(\text{Target gene})\_\text{CT}\;(\text{House keeping gene})$$

$$\text{Calculate the average}\;\Delta\text{CT of calibrator group}\;(\text{e.g.}\,\text{the control group})$$

$$\Delta\Delta\text{CT}\:=\:\Delta\text{CT}\;(\text{sample})\_\text{average}\;\,\Delta\text{CT}\;(\mathrm{calibrator}\,\mathrm{group})$$

### Field experiment and yield components

The maize SC131 grains were planted during the maize growing season in the Bani Majdul area in Kerdasa village, Giza governorate, Egypt (Lat. 30° 03 14'' N. and Long. 31° 06′ 27'' E.). The field experiment at the study location was laid out in a randomized complete block design with three replicates, and the plot size was 3 m^2^ (1 × 3 m) [31]. Each entry consisted of five rows at a distance of 50 cm. All other agricultural procedures were practiced as recommended for maize grown in such areas. Physical and chemical analyses of soil and irrigation water of the experimental site are shown in Table 2. Gamma-irradiated grains, with the same doses, were cultivated in the early summer season according to the recommended agricultural practice for the determination of the impact of gamma irradiation. After 40–50 days, half of the plants of each treatment were exposed to drought stress by reducing the number of irrigations; i.e., the radiation treatments required six irrigations until the harvesting, while the radiation plus drought treatments required only three irrigations. At maturity, the performance of yield and its attributes were determined.

**Table 1:** RT-PCR primer’s names, direction, and nucleotide sequences

Type	Gene (ID, Accesion)	Direction	5'-seq-3'
House Keeping	Polyubiquitin	Forward	GGTGGCCTCTAAATGTTCTTCTACTG
	UBQ	Reverse	CACACAGACTTCATTAGAAAGACAATCA
Water Drought	Dehydration responsive TF	Forward	ATGGAGCACGAGCAGGTGTCG
	*DREB2*	Reverse	TCAATGTTCCCAAAGCAGCAAGG
	Ethylene-Responsive TF	Forward	GCCAATTTTGTTTTCGTGCTG
	*ERF*	Reverse	CTCTCTCTCTCTTCACCTTACG
Leaf Elongation	Elongation Factor	Forward	CATGCTCTCCTTGCGTTCAC
	*EF*	Reverse	CCATACCAGGCTTGATGACAC

**Table 2:** Physical and chemical analysis of the experimental soil and chemical analysis of underground irrigation water at the study location

**a) Soil mechanical analysis**						
Total Sand (%)		Clay (%)		Silt (%)	Texture class	
36.59		34.18		29.23	clay loam	
**b) Soil chemical analysis pH = 7.16 EC = 1.04 (dS m**^**−1**^**)**						
ESP (%)		CaCO_3_ (g kg^−1^)		Gypsum (g kg^−1^)	SOM (g kg^−1^)	
13.82		25.7		16.7	22.9	
**c) Irrigation water chemical analysis pH = 6.92 EC = 0.60 (dS m**^**−1**^**)**						
Soluble cations (mmol L^−1^)				Soluble anions (mmol L^−1^)		
Ca^++^	Mg^++^	Na^+^	K^+^	CO_3_^−−^ + HCO_3_^−^	Cl^−^	SO_4_^−−^
3.17	1.34	1.25	0.33	2.92	1.28	1.89

### SDS-PAGE analysis

According to the procedure outlined by Laemmli [32] and modified by Studier [33]**,** the electrophoresis pattern of stored protein from harvested grains under treatments was examined on 10% polyacrylamide gels. Following electrophoresis, the gel was fixed and stained with Coomassie Brilliant Blue R-250 at 0.25% (w/v). Using the Gel Doc 2000 Bio-Rad system, the gel was photographed, scanned, and evaluated.

### Statistical analysis

A two-way analysis of variance (ANOVA) was used to evaluate the effect of gamma radiation and drought on maize SC131 genotype. The experimental data were analyzed using IBM SPSS software version 24 (IBM Corp., Armonk, New York, USA). Means were compared using the Duncan multiple range test at a 5% level of significance (*p* < 0.05).

## Results

### Cytological impacts of γ-radiation and drought stress

The mitotic activity in root tip cells of the maize SC131 genotype used in this study was scored as MI values (Table 3). The low γ-radiation dose of 50Gy caused significantly increased (*p* ≤ 0.05) in the MI value in maize plants compared to the control. In contrast, the 100, 150, 200 and 250Gy doses significantly decrease the MI values. Regarding drought (10% PEG) treatment, a highly significant decrease in the MI value compared to the control, but the treatment of drought combined with the dose 50Gy caused significantly increased (*p* ≤ 0.05) in the MI value when compared to drought treatment.

**Table 3:** Mitotic index, phase indices, types, and percentages of mitotic abnormalities (mean ± SD) induced by different doses of γ-irradiation alone and in combination with drought stress imposed by 10% of polyethylene glycol on root tip cells of maize SC131 genotype

Treatment	No. of dividing cells	Mitotic Index%	Phase Index%				Mitotic aberrations (%)			Total mitotic abnormalities (%)
			**Prophase**	**Metaphase**	**Anaphase**	**Telophase**	**Spindle disturbance**	**Clastogenic aberrations**	**Chromosome stickiness**	
**Control**	555	11.1 ± 0.93^a^	43 ± 1.22^b^	11.68 ± 0.87^a^	13.72 ± 1.1^a^	31.6 ± 2.54^d^	1.15 ± 0.89^e^	0.04 ± 0.01^ g^	0.18 ± 0.18^f^	1.37 ± 0.06^ g^
**γ 50 Gy**	562	11.24 ± 0.44^a^	54.43 ± 3.95^ab^	7.17 ± 0.33^ab^	6.50 ± 1.23b	31. 9 ± 3.09^d^	20.68 ± 1.94^d^	2.01 ± 0.13^e^	3.93 ± 0.11^e^	26.62 ± 0.41^f^
**γ 100 Gy**	552	11.04 ± 0.78^a^	56.14 ± 1.44^ab^	5.24 ± 0.71^b^	3.97 ± 1.4^c^	34.65 ± 1.61^c^	27.43 ± 3.17^bc^	5.58 ± 0.27^d^	5.37 ± 0.57^d^	38.38 ± 0.56^e^
**γ 150 Gy**	543	10.86 ± 0.52^a^	58.99 ± 3.61^a^	7.23 ± 0.05^ab^	2.55 ± 1.04^d^	31.23 ± 3.84^d^	25.86 ± 2.46^c^	9.76 ± 0.87^c^	8.54 ± 0.77^c^	44.16 ± 2.91^d^
**γ 200 Gy**	516	10.32 ± 0.45^ab^	57.22 ± 3.6^ab^	4.19 ± 0.8^c^	2.39 ± 0.78^d^	36.20 ± 3.71^c^	19.98 ± 1.54^d^	14.46 ± 1.64^bc^	12.78 ± 0.35^b^	47.22 ± 2.44^ cd^
**γ 250 Gy**	397	7.94 ± 0.94^c^	57.11 ± 2.72^ab^	5.78 ± 0.16^b^	2.43 ± 1.06^d^	34.68 ± 3.69^c^	30.77 ± 2.35^b^	18.04 ± 1.94^b^	11.26 ± 0.81^b^	60.07 ± 1.46^b^
**Drought (D) (10% PEG)**	423	8.46 ± 0.82^bc^	42.87 ± 2.95^c^	5.57 ± 0.86^b^	5.7 ± 0.71^b^	45.86 ± 1.48^b^	19.13 ± 3.89^d^	0.84 ± 0.51^f^	8.15 ± 0.33^c^	28.12 ± 1.14^f^
**D** + **γ 50 Gy**	443	8.86 ± 0.51^bc^	46.89 ± 3.75^b^	6.46 ± 0.59^ab^	4.39 ± 1.51^bc^	42.26 ± 5.19^bc^	30.5 ± 2.51^b^	3.74 ± 0.24^de^	10.72 ± 0.07^b^	44.96 ± 2.68^d^
**D** + **γ 100 Gy**	403	8.06 ± 0.96^bc^	56.37 ± 2.34^ab^	4.06 ± 0.3^ cd^	6.15 ± 0.85^b^	33.42 ± 2.29^d^	24.34 ± 1.77^c^	10.19 ± 0.69^c^	15.58 ± 0.55^a^	50.11 ± 3.87^c^
**D** + **γ 150 Gy**	397	7.94 ± 0.43^c^	49.25 ± 3.27^b^	5.10 ± 0.4^c^	1.38 ± 0.87^e^	44.27 ± 3.83^b^	34.62 ± 4.27^ab^	16.24 ± 2.77^b^	13.84 ± 0.83^a^	64.70 ± 3.63^b^
**D** + **γ 200 Gy**	354	7.08 ± 0.55^d^	43.96 ± 5.62^c^	2.90 ± 0.92^d^	3.62 ± 1.61^c^	49.52 ± 6.3^a^	39.62 ± 5.66^a^	28.15 ± 1.11^a^	13.32 ± 0.55^a^	81.09 ± 2.34^a^
**Significant**	–-	**	**	*	**	**	**	**	**	**
**Treatment (γ)**	–-	*	*	*	**	**	**	**	**	**
**Drought (D)**	–-	**	**	*	**	**	**	**	**	**
γ *D	–-	**	**	**	**	**	**	**	**	**

5000 cells were scored per treatment

The results were the mean of three determinations from three separate experiments. Means with different letters in the same column were significantly different at *p* ≤ 0.05 according to Duncan's multiple range test. * and ** indicate differences at *p* value < 0.05 or 0.01, respectively

**Fig. 1:**
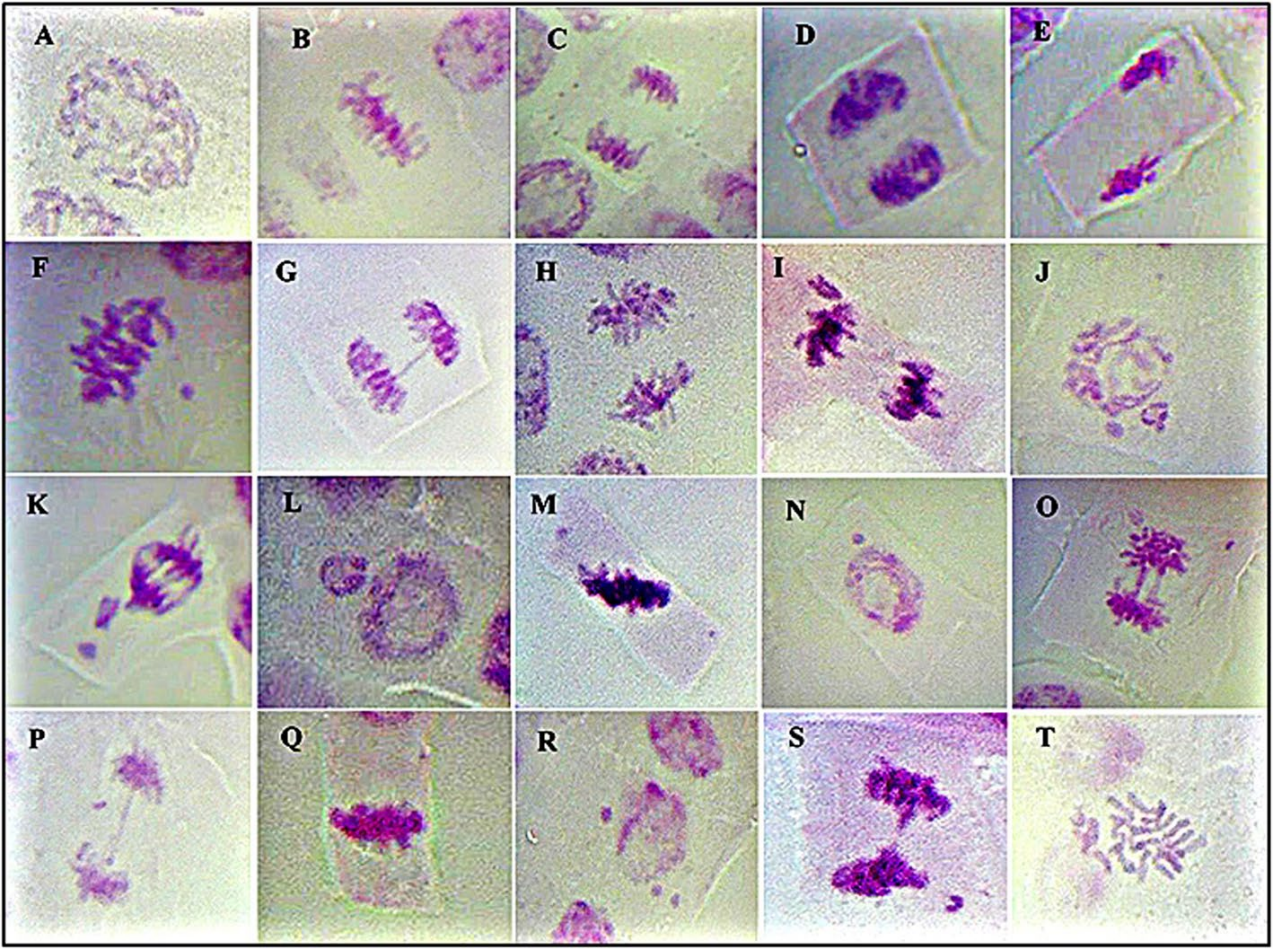
The effect of different doses of γ-irradiation alone and in combination with drought stress imposed by 10% of PEG on representative examples of normal (control) and abnormal cell divisions in maize SC131 root tips. **A**–**D** Normal prophase, metaphase, anaphase, and telophase, respectively, under control conditions (distilled water). **E**,** F** Diagonal sticky anaphase and disturbance in metaphase with non-oriented chromosome after treatment with γ (100 Gy). **G** Chromosomal Bridge in anaphase after treatment with γ (150 Gy). **H** disturbance in anaphase after treatments with γ (50 Gy). **I**,** J** Lagging chromosome in anaphase and micronuclei in prophase after treatment with γ (200 Gy). **K** multi chromosomal bridges with lagging chromosome and fragment after treatment with γ (250 Gy). **L** Micronucleus after treatment with drought (10%PEG). **M**,** N** Sticky diagonal metaphase and micronucleus after treatment with D + γ (50 Gy). **O**,** P** multi bridges with fragments and chromosomal bridge with lagging chromosomes after treatment with D + γ (100 Gy). **Q**,** R** sticky metaphase and micronuclei after treatment with D + γ (150 Gy). **S**,** T** Sticky anaphase with bridge and lagging chromosome and C-metaphase after treatment with D + γ (200 Gy)

Three major types of aberrations were identified: (a) spindle disturbance and its consequences (e.g., C-metaphase, lagging chromosome, star metaphase/anaphase, multipolar anaphase/telophase, unequal distribution, phase disturbance, and non-oriented chromosome), (b) clastogenic chromosome aberrations (e.g., bridges, fragments or breaks, micronuclei, and ring chromosomes), and (c) chromosome stickiness. All treatments, i.e., doses of γ-radiation (from 50 to 250 Gy), simulated drought (imposed by 10% PEG) and γ-radiation accompanied with drought stress induced significantly increased (*p* ≤ 0.05) in the proportion of cells showing chromosomal abnormalities. The γ-radiation dose of 50 Gy showed the lowest percentage of chromosomal abnormalities compared to the higher doses of γ-radiation, while the treatment of γ-radiation 200Gy combined with drought scored the highest percentage of chromosomal abnormalities as illustrated in Table 3. Different types of chromosomal aberrations were detected in all treatments, like diagonal sticky anaphase, disturbance in metaphase, non-oriented chromosome, chromosomal bridge, disturbance in anaphase, lagging chromosome in anaphase, micronuclei in prophase, multi-chromosomal bridges with lagging chromosome, chromosome fragments, diagonal sticky metaphase, micronucleus in interphase, and C-metaphase, as shown in Fig. 1.

**Fig. 2:**
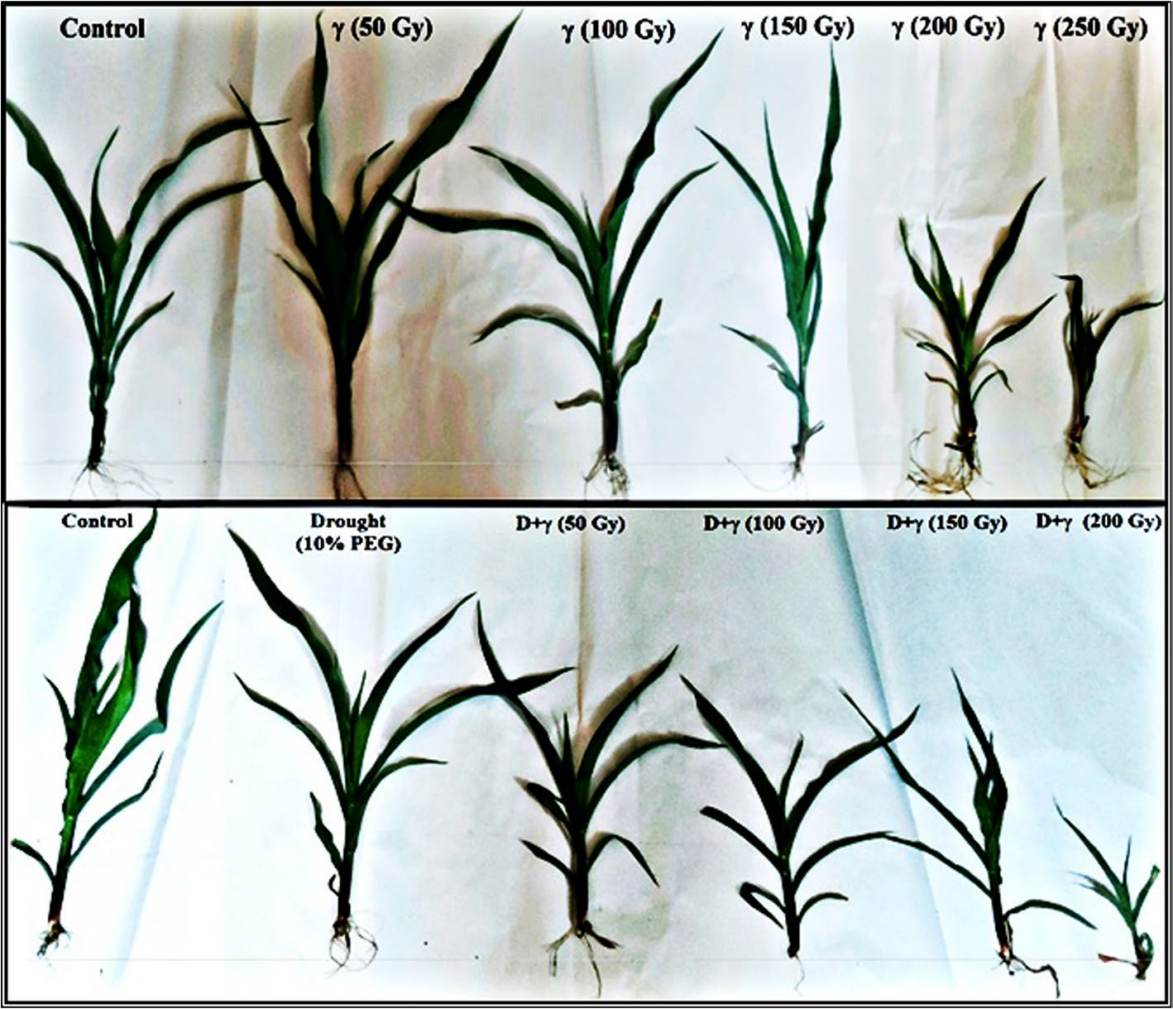
The effect of different doses of γ-irradiation alone and in combination with drought stress imposed by 10% of PEG on maize SC131 genotype seedling stages after 21 days

### The impact of γ-radiation and drought stress (10% PEG) on growth parameters

All morphological growth parameters measured, in this study, clearly indicate that the low dose of 50 Gy was significantly increased (*p* ≤ 0.05) the vegetative growth of the shoot and root lengths, fresh and dry weights, and caused a similar influence on leaf measurements at early stages of growth of 21 days after sowing compared to the control. In contrary, the high dose of 250 Gy was significantly reduced (*p* ≤ 0.05) all growth parameter measurements. For simulated drought stress treatment (imposed by 10% PEG), all growth parameters were significantly reduced (*p* ≤ 0.05) in compare to control. While, the treatment of drought combined with γ 50 Gy improved plant tolerance to drought stress by improving the measurements in compare to drought treatment alone. As for the drought treatments combined to the higher doses of γ-radiation, they were highly significantly reducing all growth parameter measurements (Table 4). Moreover, an increased sensitivity of SC131 genotype to the treatment of drought combined with γ 250 Gy was noticed and no seed germination occurred. Figure 2 illustrates variations in morphological traits of 21 days seedling stage of maize SC131 genotype after exposure to different treatments of γ-radiation doses, drought (10% PEG) and γ-radiation doses combined to drought.

**Table 4:** The effect of different doses of γ-irradiation alone and in combination with drought stress imposed by 10% of polyethylene glycol on growth parameters and plant biomass of maize SC131 genotype after 21 days of planting

Treatments	Root length (cm)	Root fresh weight (g)	Root dry weight (g)	Shoot length (cm)	Shoot diameter (cm)	Shoot fresh weight (g)	Shoot dry weight (g)	Number of leaves	Leaf surface area (cm^2^)
**Control**	30.10 ± 1.5^a^	0.51 ± 0.01^a^	0.056 ± 0.002^b^	41.40 ± 0.93^a^	1.1 ± 0.06^a^	1.54 ± 0.20^a^	0.13 ± 0.002^a^	4.8 ± 0.20^b^	18.27 ± 1.20^a^
γ **50 Gy**	31.16 ± 1.89^a^	0.54 ± 0.04^a^	0.061 ± 0.001^a^	41.58 ± 2.77^a^	1.18 ± 0.07^a^	1.59 ± 0.22^a^	0.14 ± 0.002^a^	5.0 ± 0.30^a^	18.71 ± 1.88^a^
γ **100 Gy**	28.32 ± 2.65^b^	0.5 ± 0.05^a^	0.054 ± 0.003^b^	38.32 ± 1.36^b^	0.94 ± 0.08^b^	1.41 ± 0.23^a^	0.12 ± 0.001^b^	4.6 ± 0.24^b^	17.31 ± 1.68^b^
γ **150 Gy**	20.46 ± 2.45^de^	0.35 ± 0.04^c^	0.042 ± 0.004^c^	25.6 ± 0.76^d^	0.64 ± 0.09^d^	0.7 ± 0.12^d^	0.06 ± 0.003^c^	4.6 ± 0.24^b^	7.90 ± 0.21^e^
γ **200 Gy**	18.96 ± 1.76^de^	0.3 ± 0.01^c^	0.033 ± 0.002^d^	22.2 ± 0.74^e^	0.42 ± 0.02^e^	0.57 ± 0.10^e^	0.04 ± 0.004^d^	4.4 ± 0.24^b^	7.60 ± 0.54^e^
γ **250 Gy**	14.46 ± 0.79^ g^	0.25 ± 0.01^d^	0.021 ± 0.003^e^	20.24 ± 1.04^e^	0.4 ± 0.02^e^	0.53 ± 0.05^e^	0.04 ± 0.001^d^	4.0 ± 0.32^b^	5.80 ± 0.87^f^
**Drought (D)** **(10% PEG)**	24.08 ± 1.11^d^	0.33 ± 0.05^c^	0.038 ± 0.003^d^	33.54 ± 2.06^c^	0.92 ± 0.09^b^	0.92 ± 0.15^c^	0.07 ± 0.008^c^	4.2 ± 0.20b	13.43 ± 1.58^c^
**D + **γ **50 Gy**	25.6 ± 2.43^bc^	0.45 ± 0.03^b^	0.051 ± 0.001^b^	34.24 ± 1.8^c^	1.04 ± 0.04^a^	1.11 ± 0.14^b^	0.08 ± 0.002^c^	4.4 ± 0.41^b^	13.67 ± 1.34^c^
**D + **γ** 100 Gy**	17.7 ± 2.17^e^	0.31 ± 0.04^c^	0.033 ± 0.001^d^	25.7 ± 1.98^d^	0.76 ± 0.04^c^	0.79 ± 0.1^d^	0.06 ± 0.001^c^	4.2 ± 0.21^b^	9.53 ± 1.6^d^
**D + **γ **150 Gy**	17.6 ± 2.97^e^	0.27 ± 0.04^d^	0.031 ± 0.002^d^	24.26 ± 1.19^d^	0.44 ± 0.04^e^	0.68 ± 0.03^d^	0.03 ± 0.001^e^	4.0 ± 0.32^b^	6.80 ± 0.96^e^
**D + **γ** 200 Gy**	16.0 ± 1.0^f^	0.23 ± 0.01^d^	0.026 ± 0.001^e^	16.00 ± 1.0^f^	0.24 ± 0.04^f^	0.25 ± 0.04^f^	0.02 ± 0.003^f^	3.2 ± 0.22^c^	5.58 ± 0.51^f^
**Significant**	**	**	**	**	**	**	**	*	**
**Treatment (γ)**	**	**	**	*	**	**	**	ns	**
**Drought (D)**	**	**	**	**	**	**	**	ns	**
γ *D	**	**	**	**	**	**	**	ns	**

The values are the means of five replicates with standard deviation (± SD). Values followed by the same letter (s) are not significantly different according to Duncan's multiple range test at *P* ≤ 0.05 level.*and **indicate differences at a *p*-value < 0.05 or 0.01, respectively

### Impact of γ-radiation and drought stress (10% PEG) on Gene expression using RT-PCR

Gene expression quantification is a commonly utilized technique in molecular genetics for measuring gene expression levels by referencing a housekeeping gene, thereby assessing its molecular status, whether up-regulated or down-regulated. Additionally, qPCR can distinguish relative gene expression between the same genotype under different conditions, like control versus treatment. Three genes were measured and identified as transcription factors, known as *DREB2*, *ERF* and *EF*. Both *DREB2* and *ERF* genes were previously reported as molecular markers for abiotic stress tolerance. RNA conversion into cDNA used reverse transcription to quantitatively analyze the gene expression (qPCR) of the *DREB2*, *ERF* and *EF* genes for tested genotype, along with the ubiquitin as a house-keeping gene as an indicator to know the extent of the change in the gene expression under: 1) drought (10% PEG), 2) drought combined with γ-radiation lowest dose of 50 Gy, and 3) drought combined with γ-radiation highest dose of 200 Gy. After the Real-Time PCR run, the obtained CT values were used to estimate ΔCT and the fold change of gene expression of *DREB2*, *ERF* and *EF* genes using subtractive equations.

**Fig. 3:**
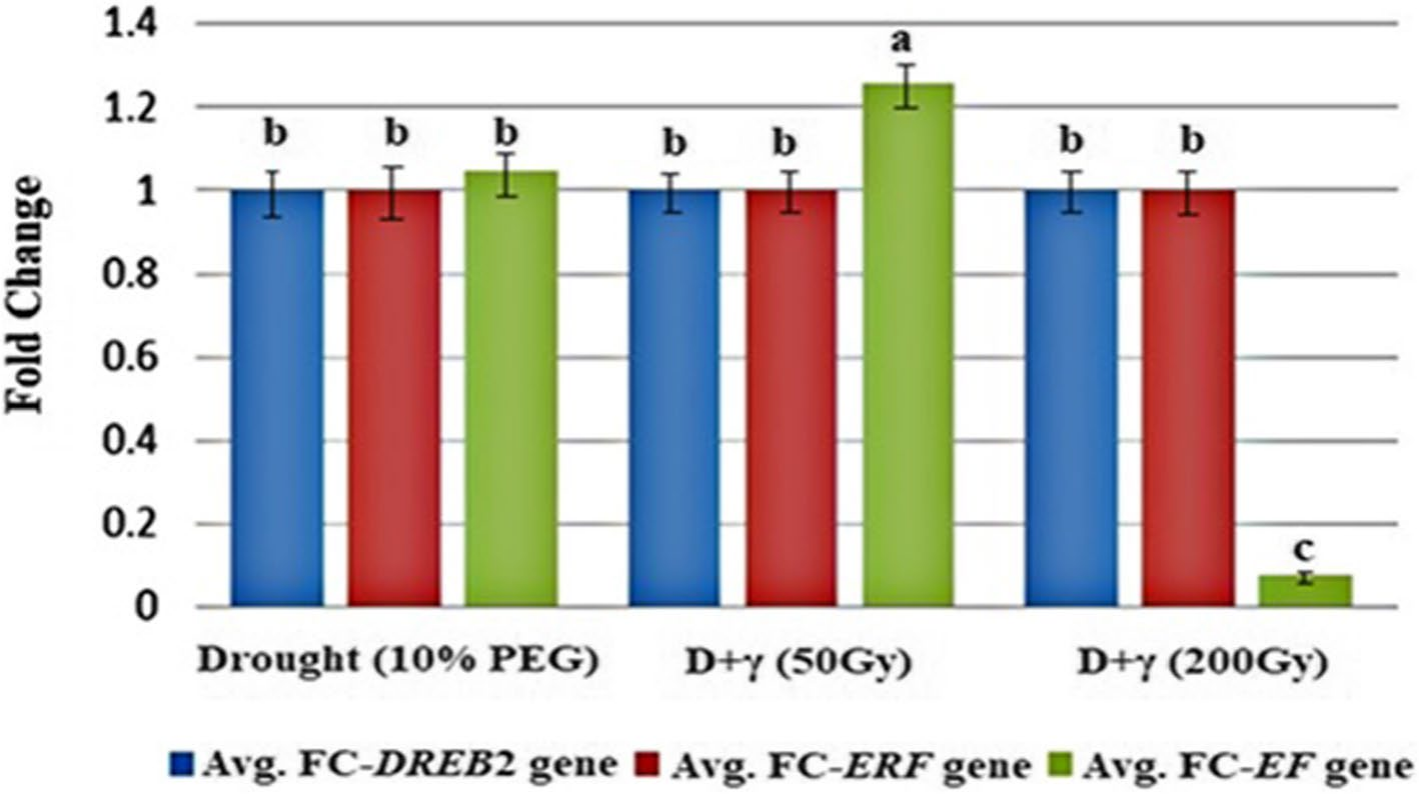
The effect of different doses of γ-irradiation alone and in combination with drought stress imposed by 10% of PEG on the relative expression (avg. FC) of *DREB2* gene, *ERF* gene, and *EF* gene in maize SC131 genotype. The results were the mean of three determinations from three separate experiments. The bars represent the mean ± SD values. Means with different letters in the same column were significantly different at *p* ≤ 0.05 according to Duncan's multiple range test

By extracting the CT values for all the triplicated samples, the CT values were observed as: 20 for the Ubiquitin (UBQ) gene (house-keeping gene as an indicator), 27 for the *DREB2*, 26 for the *ERF*, and ranged from 25 to 29 for the *EF* transcription factors. The CT values of the transcription factors were subtracted from the CT values of the house-keeping gene independently to define the changes in CT values (ΔCT). The ΔCT and ∆∆CT values were calculated to estimate the relative rate of change in the gene expression (fold change) for each replicate. The fold change average of the triplicates underwent calculation to represent each genotype per quantified genes. In both *DREB2* and *ERF* transcription factors the calculated fold change averages were 1.00 with no significant differences for the three tested treatments, while the calculated fold change averages of *EF* transcription factors showed significant differences among the three treatments (drought, drought + dose 50 Gy, and drought + dose 200 Gy) of 1.0499, 1.2599 and 0.0787, respectively (Table 5, Fig. 3).

**Table 5:** The CT values, delta-CT and ratio of relative expression (Fold change FC) of *DREB*, *ERF* and *EF* genes in maize SC131 genotype

Treatment	Replicate	UBQ	*DREB* (CT)	*ERF* (CT)	*EF* (CT)	dCT-*DREB*	dCT-*ERF*	dCT-*EF*	ddct-*DREB*	ddct-*ERF*	ddct-*EF*	Fold change-*DREB*	Fold change-*ERF*	Fold change-*EF*	Avg.FC-*DREB*	Avg.FC-*ERF*	Avg.FC-*EF*
**Drought (10% PEG)**	R1	20	27	26	26	7	6	6	0	0	0.667	1.00	1.00	0.629961	1.00	1.00	1.0499
	R2	20	27	26	25	7	6	5	0	0	−0.333	1.00	1.00	1.259921			
	R3	20	27	26	25	7	6	5	0	0	−0.333	1.00	1.00	1.259921			
**Drought + **γ **50Gy**	R1	20	27	26	25	7	6	5	0	0	−0.333	1.00	1.00	1.259921	1.00	1.00	1.2599
	R2	20	27	26	25	7	6	5	0	0	−0.333	1.00	1.00	1.259921			
	R3	20	27	26	25	7	6	5	0	0	−0.333	1.00	1.00	1.259921			
**Drought + **γ **200Gy**	R1	20	27	26	29	7	6	9	0	0	3.667	1.00	1.00	0.078745	1.00	1.00	0.0787
	R2	20	27	26	29	7	6	9	0	0	3.667	1.00	1.00	0.078745			
	R3	20	27	26	29	7	6	9	0	0	3.667	1.00	1.00	0.078745

### The impact of γ-radiation and drought stress on yield attributes

The effect of γ-radiation doses as well as the effect of γ-radiation combined with drought stress imposed by reducing the number of irrigations were examined under field conditions. The results of this experiment indicated that the low dose of γ-radiation (50 Gy) significantly enhanced (*p* ≤ 0.05) all tested yield traits (plant height, cob length, cob dry weight, number of kernel rows/ear, number of kernels (seeds)/row, number of kernels/cob, weight of kernels on cob, weight of 1000 grains and grain yield/plant) compared to the control group. On the other hand, when γ-radiation doses increased, these traits significantly decreased (*p* ≤ 0.05) in comparison to the control group (Table 6).

**Table 6:** The effect of different doses of γ-irradiation alone and in combination with drought stress on yield components and their attributes of maize SC131 genotype after 115 days of planting

	**Plant height** **(m)**	**Cob length** **(cm)**	**Cob dry weight** **(g)**	**No., of Kernel rows/Ear**	**Number of Kernels (seeds)/row**	**Number of Kernels/Cob**	**Weight of Kernels on Cob** **(g)**	**Weight of 1000 grains** **(g)**	**Grain Yield/Plant (g)**
Control	2.67 ± 0.10^a^	21.58 ± 0.58^b^	37.58 ± 0.44^b^	14.6 ± 0.89^a^	37.14 ± 0.7^a^	541.4 ± 9.55^b^	179.04 ± 2.21^b^	357.02 ± 4.45^b^	301.75 ± 8.01^b^
γ 50Gy	2.81 ± 0.23^a^	23.56 ± 0.18^a^	45.71 ± 0.42^a^	14.8 ± 1.79^a^	38.78 ± 0.5^a^	569.6 ± 6.77^a^	209.91 ± 2.92^a^	382.77 ± 2.72^a^	368.69 ± 8.60^a^
γ 100Gy	2.46 ± 0.17^b^	20.82 ± 0.77^c^	36.32 ± 0.88^b^	13.8 ± 1.1^b^	36.25 ± 0.64^b^	536.2 ± 11.47^b^	169.56 ± 8.14^c^	354.12 ± 3.51^b^	271.13 ± 4.49^c^
γ 150Gy	2.31 ± 0.10^c^	20.38 ± 0.25^c^	34.44 ± 0.15^c^	13.6 ± 2.19^b^	29.73 ± 0.72^e^	409.0 ± 14.37^e^	143.07 ± 7.05^d^	339.47 ± 2.76^c^	189.55 ± 5.95^f^
γ 200Gy	2.24 ± 0.05^d^	19.1 ± 0.60^d^	30.7 ± 0.20^d^	12.4 ± 0.89^c^	15.04 ± 0.60^g^	187.4 ± 18.18^g^	58.30 ± 4.61^h^	329.06 ± 2.16^d^	118.88 ± 2.69^i^
γ 250Gy	2.03 ± 0.17^e^	21.3 ± 0.48^b^	29.2 ± 0.49^de^	11.1 ± 1.41^d^	12.78 ± 0.30^h^	153.0 ± 18.32^h^	52.95 ± 6.51^i^	313.99 ± 3.85^e^	69.02 ± 2.19^j^
Drought (D)	2.30 ± 0.13^c^	20.8 ± 0.46^c^	34.17 ± 0.53^c^	14.0 ± 1.41^a^	32.53 ± 0.58^d^	421.67 ± 10.73^d^	135.06 ± 3.36^e^	341.62 ± 1.87^c^	209.29 ± 2.75^e^
D + γ 50Gy	2.55 ± 0.03^b^	22.94 ± 0.60^a^	42.9 ± 0.36^a^	14.0 ± 2.24^a^	34.78 ± 0.79^c^	433.1 ± 12.03^c^	140.73 ± 3.25^d^	356.49 ± 2.56 ^b^	258.03 ± 3.51^d^
D + γ 100Gy	2.25 ± 0.17^d^	20.92 ± 0.93^c^	34.6 ± 0.60^c^	14.0 ± 1.41^a^	28.59 ± 0.74^e^	404.6 ± 17.26^e^	109.45 ± 4.17^f^	336.42 ± 4.02^cd^	173.53 ± 0.55^g^
D + γ 150Gy	2.25 ± 0.14^d^	20.4 ± 0.72^c^	31.64 ± 0.34^d^	13.2 ± 1.1^b^	22.34 ± 0.63^f^	335.4 ± 8.20^f^	97.10 ± 2.01^g^	329.65 ± 1.71^d^	135.08 ± 2.33^h^
D + γ 200Gy	2.14 ± 0.18^e^	17.9 ± 0.95^e^	26.81 ± 0.21^e^	11.6 ± 1.67^cd^	11.34 ± 0.20^h^	137.6 ± 8.12^h^	48.35 ± 3.54^j^	321.67 ± 2.01^d^	61.81 ± 1.02^k^
Significant	**	**	**	**	**	**	**	**	**
Treatment (γ)	**	**	**	*	**	**	**	**	**
Drought (D)	**	**	**	*	**	**	**	*	**
γ *D	**	**	**	**	**	**	**	**	**

The values are the means of five replicates with standard deviation (± SD). Values followed by the same letter (s) are not significantly different according to Duncan's multiple range test at *P* ≤ 0.05 level. *and **indicate difference at *p* value < 0.05 or 0.01, respectively

**Fig. 4:**
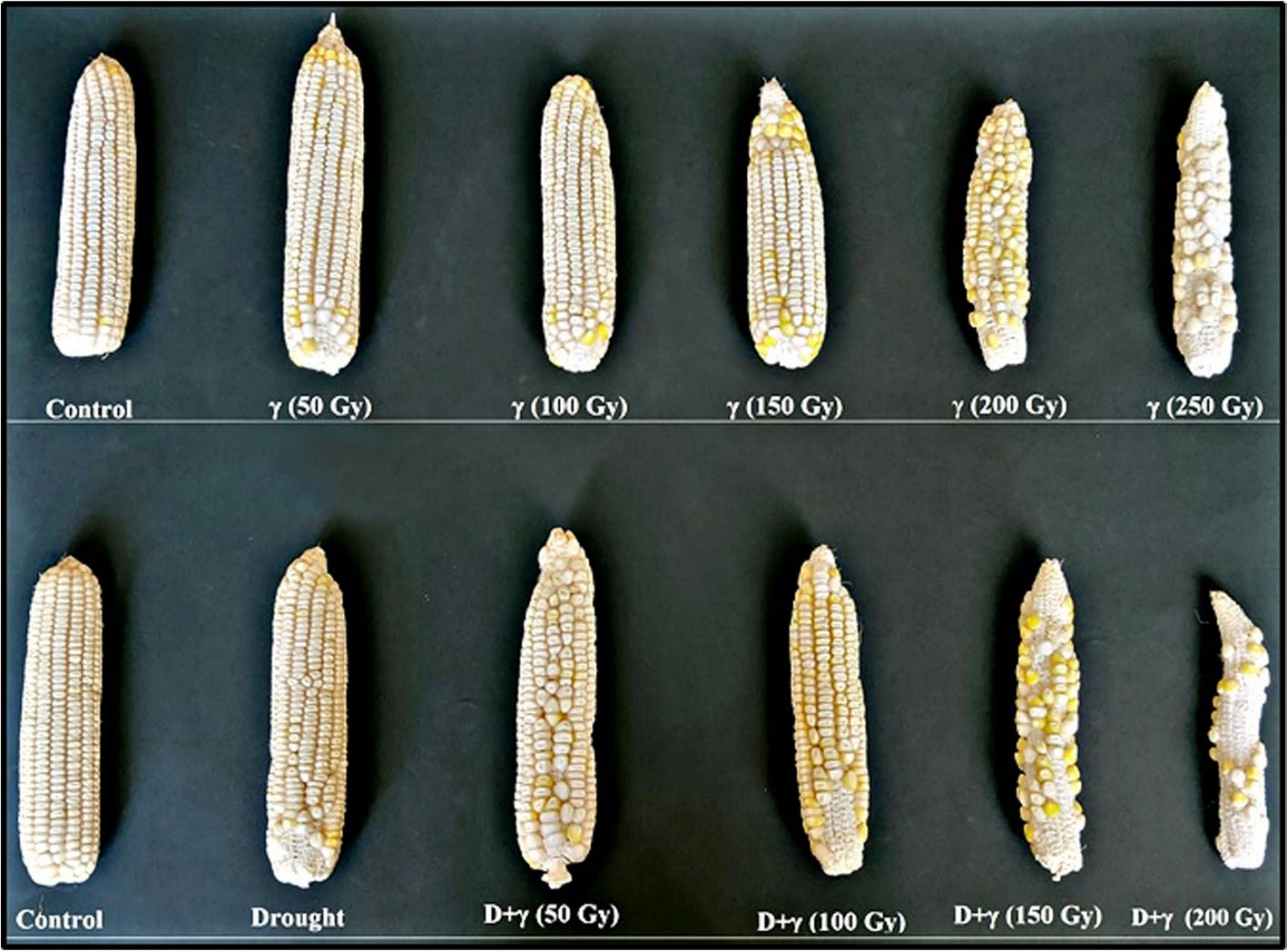
The effect of different doses of γ-irradiation and γ-irradiation combined with drought stress on cobs of maize SC131 genotype

Furthermore, with the drought treatment (D), the mean values of all tested yield attributes decreased significantly when compared to the control group. However, combining drought with a low dosage of γ-radiation (50 Gy) improved yield attributes more than drought alone. Drought treatments with the other high doses of γ-radiation (D + 100, D + 150, and D + 200 Gy) resulted in significant decreases in all yield traits compared to the control group (Table 6, Fig. 4). Moreover, an increased sensitivity of SC131 genotype to the treatment of drought combined with γ 250 Gy was noticed and no seed germination occurred.

**Fig. 5:**
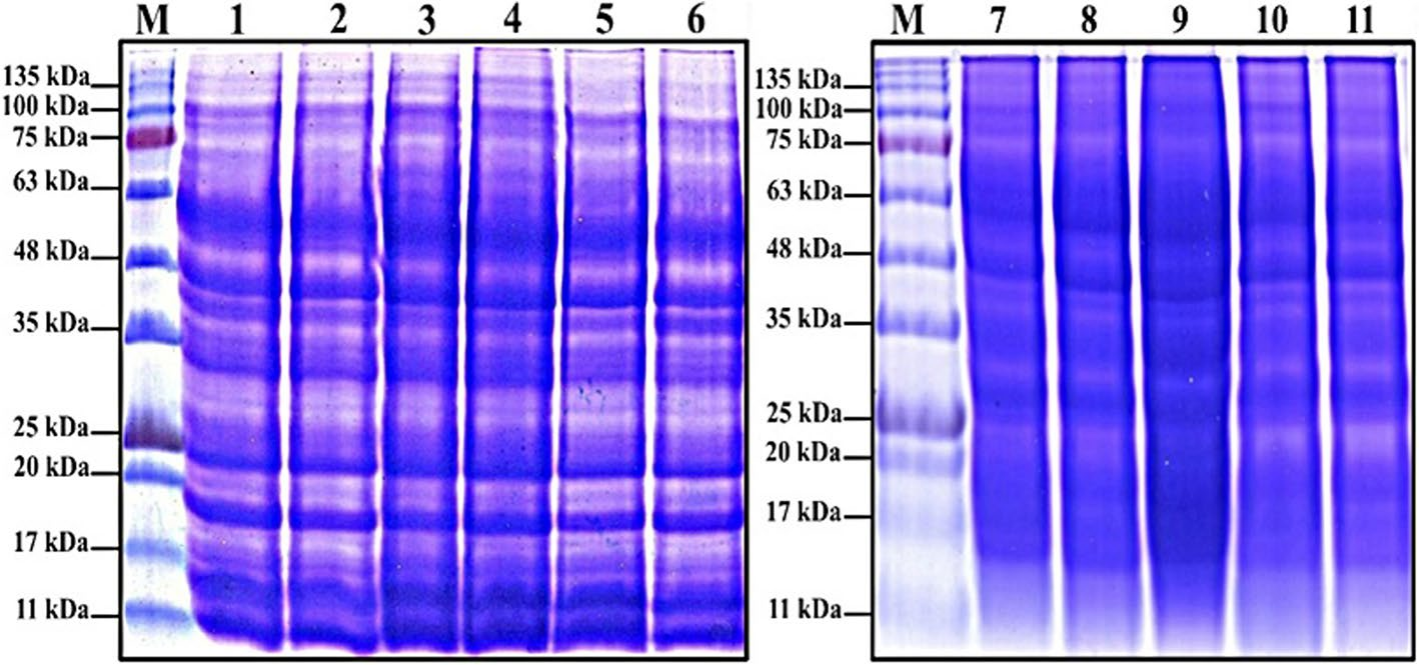
The effect of different doses of γ-irradiation alone and in combination with drought stress on SDS banding pattern of yielded grains protein of the maize SC131 genotype. M (protein marker), Lanes 1–11: Control1, γ 50, γ 100, γ 150, γ 200, γ 250Gy, Drought, D + γ 50, D + γ 100, D + γ 150, D + γ 200Gy, respectively

### The impact of γ-radiation and drought stress on total seed storage proteins

Figure 5 illustrates the SDS-PAGE banding pattern of the resultant seed storage protein for the maize SC131 genotype, under varying doses of γ-irradiation and when irradiation is combined with drought treatment. From the obtained results, it is clear that 17 bands were recorded in seed protein patterns (Table 7). Such bands were detected at approximately molecular mass ranging between 155.74 and 10.58 kDa and relative mobility (Rf) values between 0.042 and 0.963. Eight out of the scored bands were recognized as monomorphic, while the remaining nine bands were considered polymorphic ones. The monomorphic bands have molecular masses of 155.74, 90.23, 43.85, 38.24, 33.25, 27.06, 18.57, and 15.45 kDa, while the polymorphic bands have molecular masses of 124.68, 101.62, 80.27, 62.92, 54.31, 31.62, 21.47, 12.77, and 10.58 kDa.

**Table 7:**
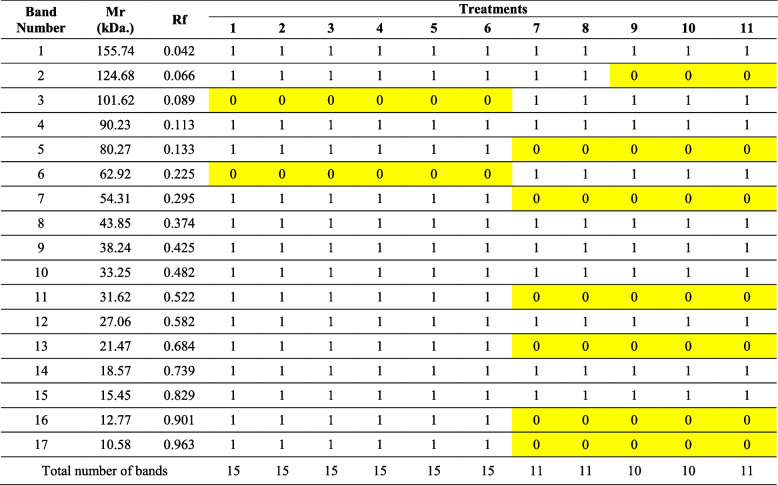
The effect of different doses of γ-irradiation alone and in combination with drought stress on the molecular mass (Mr.) in kilo-Daltons (kDa) and the Retention factor (Rf) of the produced SDS-PAGE of seed protein bands and their presence (1) or absence (0) in the maize SC131 genotype

*Lanes 1–11: Control, γ 50, γ 100, γ 150, γ 200, γ 250Gy, Drought (D), D + γ 50, D + γ 100, D + γ 150, D + γ 200Gy, respectively. Yellow highlights indicated the positive and negative markers

From these polymorphic bands, there are two bands with molecular masses of 101.62 and 62.92 kDa that appeared and were characteristic for drought treatment (D) as well as drought accompanied with γ-irradiation doses (D + γ 50, D + γ 100, D + γ 150, and D + γ 200 Gy), while not found in the control group and γ-irradiation doses (γ 50, γ 100, γ 150, γ 200, γ 250 Gy). These two bands could be used as positive biochemical markers for drought stress. On the other hand, there are six polymorphic bands with molecular masses of 80.27, 54.31, 31.62, 21.47, 12.77, and 10.58 kDa that appeared and were characteristic for control and γ-irradiation doses (γ 50, γ 100, γ 150, γ 200, and γ 250 Gy), while not found in the drought treatment (D) as well as drought accompanied with γ-irradiation doses (D + γ 50, D + γ 100, D + γ 150, and D + γ 200 Gy). These six bands could be used as negative biochemical markers for drought stress in maize SC131 genotype.

## Discussion

The gamma radiation biological effect is based on the interaction with atoms or molecules in the cell, particularly water, to produce free radicals [10]. These radicals can damage or modify important components of plant cells and have been reported to affect differently the morphology, anatomy, biochemistry, and physiology of plants depending on the radiation dose [34]. These effects include changes in the plant cellular structure and metabolism e.g., dilation of thylakoid membranes, alteration in photosynthesis, modulation of the anti-oxidative system, and accumulation of phenolic compounds [11]. Gamma radiations have enormous potential for diverse agricultural uses, as indicated by their interaction at the crop, plant, tissue, and cell levels. Gamma radiation interacts with plant biomolecules through reduced production, in vivo immobilization, or degradation, causing a decrease or increase in the level of respective molecules and resulting in cytological, agro-morphological, biochemical and molecular changes that affect plant growth, vigor, and yield, stored agriproduct as well as tolerance of abiotic stresses [35].

The findings from this study highlight the complex relationship between γ-radiation exposure and mitotic activity in root tip cells of the maize SC131 genotype. The observed slight increase in mitotic index (MI) at the low γ-radiation dose of 50 Gy (11.24 ± 0.44), compared to the control (11.1 ± 0.93), suggests a potential stimulatory effect of this low radiation dose on cellular division. This aligns with previous research indicating that low doses of gamma irradiations can induce adaptive responses in plant cells, potentially enhancing their growth and resilience, and stimulating the production of few reactive oxygen species (ROS) that mediate the acceleration of cell cycle entry to G0/G1 leading to enhanced plant cell cycle machinery [36]. In contrast, the significant decrease in MI values at higher doses (100, 150, 200, and 250 Gy) indicates that excessive radiation exposure is detrimental to cellular division. The inhibitory effects of radiation on the MI indicated that gamma rays have mutagenic effects on the root tips of maize. High doses of ionized radiation induce DNA double-strand breaks which trigger genetic instability if persisted without repair and lead to gross chromosomal rearrangements to alleviate the destabilizing effect of the radiation [37]. The frequency of chromosomal abnormalities was dose dependent and its percentage varied among treatments. Three major classes of aberrations were frequently observed; (a) Spindle disturbance and its consequences (e.g., C-metaphase, lagging chromosome, phase disturbance and non-oriented chromosome); (b) Clastogenic aberrations on chromosomes (e.g., bridges, fragments or breaks, micronuclei and ring chromosomes); (c) Chromosome stickiness. The abnormalities reported here are mainly due to a clastogenic action that may have cause breakage and reunion of chromosomes. These findings are consistent with the broader literature on the effects of high radiation doses on plant physiology, where increased stress levels often result in impaired growth and reduced cell division [9]. Furthermore, the effects of drought stress, induced by 10% PEG treatment, resulted in a marked decrease in MI (8.46 ± 0.82) compared to the control. This reduction underscores the negative impact of drought conditions on mitotic activity, likely due to osmotic stress and subsequent cellular responses that limit cell division [38]. Interestingly, the combination of drought treatment with the low γ-radiation dose (50 Gy) led to a slight increase in MI (8.86 ± 0.51) relative to drought treatment alone. This suggests that low-level radiation may mitigate some of the adverse effects of drought stress, possibly through mechanisms that enhance cellular repair or adaptability under stress conditions [39]. On the other hand, in the combination of drought treatment with higher doses of gamma radiation, several types of chromosomal abnormalities were seen together with doses and drought (10% PEG). Here, laggards, broken chromosomal bridges, and anaphase with multiple chromosome bridges were identified alongside clastogenic abnormalities. This result showed that gamma rays will be able to be used to create new mutants in the breeding of maize plants. This finding could have implications for developing strategies to improve crop resilience in environments subjected to drought by exposure to appropriate doses of gamma radiation.

The results of this study provide compelling evidence regarding the impacts of gamma radiation on morphological growth parameters in the maize SC131 genotype. Notably, the low dose of 50 Gy significantly enhanced various growth metrics, including shoot and root lengths, fresh and dry weights, and leaf measurements at seedlings of 21 days after sowing. The enhanced effect of low doses of irradiation may be the result of a “radiation hormesis” due to transfer of energy to cellular atoms practically, hydrogen (H), carbon (C), oxygen (O), nitrogen (N) and phosphorus (P) that may lead to stimulating effect on the physiological reactions in living cells including, cell division and growth [40]. However, low doses or intensities of stress exposure can be beneficial for plant growth and yield. Such an effect is known as hormesis or eustress, a dose–response phenomenon of growth stimulation after the application of low doses of adverse factors. In contrast, high doses of these factors induce growth inhibition or can even be lethal [40]. The stimulation of growth and development of crops using ionizing radiation (radiation hormesis) has been reported by many research groups. However, specific genes contributing to the radiation stimulation of plant growth are largely unknown. Kazakova et al. [41] studied the impact of low-dose γ-irradiation on barley seed growth dynamics and gene expression in a greenhouse experiment. They found candidate genes (PM19L-like, CML31-like, and AOS2-like) are significant in radiation hormesis throughout ontogeny, aligning with plant growth dynamics and yield parameters. They suggested a new level of radiation hormesis effect execution. Hormetic effects can expand the ability of researchers to mitigate the adverse outcomes of climate change on crop production [41]. In stark contrast, the high doses of gamma radiation (100, 150, 200, and 250 Gy) resulted in significant reductions in all growth parameters measured. This decline highlights the detrimental effects of excessive radiation, which can lead to physiological stress and impair cellular functions. Studies have shown that high radiation levels often induce oxidative stress that exceeds the capacity of cellular antioxidant defenses to remove stress, causing cellular damage and inhibiting growth [42]. The observed reduction in growth is consistent with existing literature indicating that higher radiation doses can overwhelm plant defense mechanisms [17]. On the other hand, the simulated drought stress, imposed through 10% PEG treatment, uniformly decreased all measured growth parameters compared to the control. This is indicative of the adverse effects of osmotic stress, where limited water availability restricts cellular processes and overall plant development [43, 44]. However, the combination of drought stress with the low gamma radiation dose of 50 Gy led to improved growth measurements compared to drought treatment alone. This suggests that low levels of radiation may enhance plant resilience to drought stress, possibly through the activation of protective mechanisms that improve water use efficiency and promote cellular repair [17]. Conversely, the combination of drought stress with higher doses of γ-radiation, significantly reduced growth parameters, indicating increased sensitivity of the SC131 genotype to these stressors. The absence of seed germination under the 250 Gy combined with drought treatment further underscores the compounded negative effects of high radiation and drought conditions, likely overwhelming the plant’s stress response mechanisms [45]. The interpretation of this lethal effect is due to several reasons: 1. Gamma radiation has high energy and can penetrate the cells of maize, causing damage to their DNA. The radiation can break the DNA strands, leading to mutations, chromosomal aberrations, and genomic instability. In high doses, this damage overwhelms the plant's repair mechanisms, leading to cell death or irreversible mutations that prevent the plant from growing or reproducing. 2. Gamma rays also cause direct damage to various cellular components like proteins, lipids, and other vital molecules. This disrupts essential metabolic processes, causing cell death. The radiation generates reactive oxygen species (ROS) that can further damage cell membranes, enzymes, and other crucial structures, leading to cell dysfunction and eventual death. 3. The breakdown of molecules within the plant due to irradiation can produce toxic byproducts. These byproducts, such as free radicals and other reactive compounds, can further harm cellular functions and tissues, contributing to the lethal effects [46]. Overall, these findings highlight the complex interplay between radiation exposure and drought stress in influencing vegetative growth in maize. Understanding these interactions is crucial for developing strategies to enhance crop resilience in environments prone to water scarcity. The previously findings of Koutoua et al. [47] and Kiani et al. [48] were in harmony with these results.

This study also aimed at quantify the expression of three transcription factor genes *DREB2, ERF*, and *EF* under different doses of γ-radiation and drought. The quantification was facilitated by the use of a housekeeping gene (Ubiquitin) as a reference, ensuring the accuracy of the expression measurements across different treatments [26]. Our findings revealed that the *DREB2* and *ERF* transcription factors maintained a constant fold change average of 1.00 across all treatments, indicating no significant modulation of their expression under the tested stress conditions. This result aligns with previous studies suggesting that *DREB2* and *ERF* genes are stable markers for abiotic stress tolerance, as they may function more as regulatory hubs than as direct responders to acute stress signals [49]. Their consistent expression under stress could imply that these genes are constitutively active in the tested maize SC131 genotype, especially since it is recorded in seed bank of ARC as a drought-tolerant genotype, and they serve as a baseline for stress tolerance mechanisms rather than fluctuating in response to immediate environmental changes, and this is in agreement with Zhang et al. [50] and Wu et al. [51]. Conversely, the *EF* transcription factor exhibited notable variations in gene expression across treatments, with fold change averages of 1.0499, 1.2599, and 0.0787 for drought, drought with 50 Gy γ-radiation, and drought with 200 Gy γ-radiation, respectively. These significant differences suggest that the *EF* gene may play a more dynamic role in responding to combined stresses. The increased expression under moderate stress (50 Gy) could indicate an adaptive response, while the drastic drop in expression under the highest dose of radiation may reflect a detrimental effect, possibly due to radiation-induced cellular damage or metabolic disruptions. The differential expression of the *EF* transcription factor highlights the complexity of stress responses in plants [52]. While *DREB2* and *ERF* may represent stable elements of the stress response framework, *EF* appears to be more context-dependent, suggesting that its role could be linked to specific stress signaling pathways that require further exploration [53]. This variation in expression levels may also indicate a threshold effect where low to moderate stress enhances expression and potential tolerance mechanisms, while excessive stress levels lead to negative outcomes. In conclusion, our results underscore the importance of distinguishing between different transcription factors when assessing plant responses to abiotic stress such as drought. The stable expression of *DREB2* and *ERF* suggests their roles as fundamental components of the drought stress response, and this is in harmony with the findings of Nakashima, et al. [54] and Hassan, et al. [26], while the dynamic nature of *EF* indicates a nuanced role influenced by specific environmental conditions, and this is consistent with the results of Gu, et al. [52]. Future studies should investigate the mechanistic pathways governing the differential responses of these transcription factors, particularly in the context of combined stressors, to better understand the intricate regulatory networks involved in abiotic stress tolerance.

Also, this study examined the effects of γ-radiation doses and their interaction with drought stress on yield attributes in maize SC131 genotype under field conditions. The results indicate significant differences in yield traits across the treatments, with low-dose γ-radiation (50 Gy) markedly enhancing various parameters compared to the control group. Specifically, the mean values for plant height, cob length, cob dry weight, and other yield attributes were higher in plants exposed to 50 Gy, supporting the concept of radiation hormesis, where low doses of radiation can stimulate growth and productivity [55]. The observed increase in yield traits under the 50 Gy treatment suggests that low levels of γ-radiation may promote beneficial physiological responses, such as enhanced nutrient uptake and improved metabolic activity [14]. This is in harmony with previous findings that indicate low doses of radiation can stimulate adaptive responses in plants, leading to increased growth and resilience [56, 57]. Conversely, the increase in γ-radiation doses beyond 50 Gy resulted in a steady decline in yield traits. High doses of radiation have been documented to induce oxidative stress and damage cellular structures, ultimately impairing plant growth and productivity [55]. This decline underscores the detrimental effects of excessive radiation exposure on maize plants, consistent with studies that report reduced yield parameters in response to high radiation levels [55]. The simulated drought stress treatment significantly decreased all tested yield attributes compared to the control, which is consistent with the well-established effects of drought on plant physiology. Drought stress can lead to reduced photosynthesis, impaired nutrient transport, and overall lower biomass production [58]. However, the combination of drought with low-dose γ-radiation (50 Gy) improved yield attributes compared to drought treatment alone. This finding suggests that low doses of γ-radiation may enhance drought tolerance in maize, potentially through mechanisms that bolster stress response pathways, and this result is in agreement with Katiyar, et al. [59] and Romero-Galindo, et al. [60]. In contrast, the combination of drought stress with higher doses of γ-radiation (D + 100, D + 150, and D + 200 Gy) resulted in significant reductions in yield traits, indicating that the adverse effects of drought may be exacerbated by high radiation levels. The increased sensitivity of the SC131 genotype to the treatment of drought combined with 250 Gy, where no seed germination occurred, further emphasizes the negative impact of excessive radiation and drought conditions. This observation aligns with previous research highlighting the compounding effects of abiotic stressors on crop performance [61, 62].

The investigation into the effects of γ-radiation and drought stress on total seed storage proteins in the maize SC131 genotype yielded significant insights into the biochemical responses of maize under stress conditions. The SDS-PAGE analysis revealed a total of 17 protein bands, with molecular masses ranging from 155.74 to 10.58 kDa, and this is in agreement with results of Hasan and AL-Musawi [21]. The differentiation between monomorphic and polymorphic bands provides a deeper understanding of how these stressors influence protein expression patterns. Notably, eight monomorphic bands and nine polymorphic bands were identified. The presence of polymorphic bands indicates a robust adaptive response to environmental stresses, as previously reported in various studies [63]. Specifically, the bands with molecular masses of 101.62 and 62.92 kDa emerged exclusively under drought and when drought combined with varying doses of γ-irradiation. These findings suggest that these protein bands could serve as biochemical markers for drought stress, reinforcing findings by Yang, et al. [64]**,** who identified similar proteins linked to drought tolerance in maize. Conversely, the six polymorphic bands that appeared in the control and γ-irradiation treatments but were absent under drought conditions suggest a distinct biochemical response to drought stress. The identification of these negative markers aligns with the hypothesis that drought induces a specific set of stress-responsive proteins [64]. This differentiation in banding patterns underscores the complexity of maize's adaptive mechanisms, where certain proteins are upregulated in response to γ-irradiation while others are suppressed under drought conditions. The molecular mass ranges observed in this study provide critical insights into the functional roles of these proteins. The larger proteins may be involved in structural and storage functions, while the smaller proteins could be implicated in signaling pathways or stress responses [35]. The variation in band intensity further highlights the dynamic nature of protein expression in response to environmental stresses, a concept supported by recent research demonstrating the role of post-translational modifications in stress adaptation [65]. Certain proteins implicated in essential survival mechanisms are revealed by the SDS-PAGE results pertaining to drought stress. These consist of: 1. Dehydrins that prevent protein damage and dehydration in cells, 2. Antioxidant enzymes that help reduce oxidative stress brought on by reactive oxygen species (ROS), 3. Aquaporins that control water flow to reduce water loss, 4. Osmoprotectants that preserve osmotic balance; they stabilize cellular structures in the presence of water deficiency. These proteins imply cellular adaptation, defense, and survival tactics during drought stress, such as metabolic reprogramming, osmotic control, and oxidative damage repair [66]. The findings of this study provide valuable biochemical markers for assessing drought stress in maize and contribute to the understanding of how γ-radiation influences seed storage protein profiles. Further investigation, such as functional assays or gene expression analysis, could deepen the understanding of these processes and their contributions to drought tolerance.

## Conclusion

Induced mutagenesis and radiation hormesis have emerged as essential fields of plant breeding to address global food security challenges, offering tools to enhance crop productivity, resistance, and nutritional value. Among the mutation induction tools, the physical mutagen gamma rays hold promise for efficient mutation induction. Gamma radiation can supplement conventional plant breeding by boosting variety and providing particular trait improvements without drastically affecting crop phenotypes. Gamma rays have been successfully used to promote genetic variability in plant breeding for a variety of crops, including maize crops. In this work, we suggested that exposing seeds to gamma radiation, with a specific dose of 50 Gy, before planting could help crops withstand drought stress. It is so critical to understand the fundamental mechanism governing plant response to gamma radiation. Cytological, agro-morphological, biochemical, and molecular analyses also suggested that radiations have numerous applications; however, several of these potential applications in agriculture in general and stress tolerance in particular are still to be studied and exploited. The findings of this study shed light on the effective use of gamma radiation in accelerating the establishment of potentially promising parent lines, which can aid hybridization attempts to produce improved maize varieties that can withstand the environmental stresses and climate change. These data are necessary to create further genetically edited maize lines with improved yield and stress tolerance using SC131 as a parent cultivar.

## Data Availability

The data sets generated and analyzed in this study are available from the corresponding author on reasonable request.

## References

[1] Sleper DA, Poehlman JM. Breeding field crops. 5th ed. UK: Blackwell Publishing Ltd.; 2006. p. 277–81.

[2] Ahmad Z, Waraich EA, Rehman MZ, Ayub MA, Usman M, Alharby H, El Sabagh A. Foliar application of phosphorus enhances photosynthesis and biochemical characteristics of maize under drought stress. Phyton. 2021;90(2):503–14.

[3] Erenstein O, Jaleta M, Sonder K, Mottaleb K, Prasanna BM. Global maize production, consumption and trade: Trends and R&D implications. Food Security. 2022;14(5):1295–319.

[4] Sah RP, Chakraborty M, Prasad K, Pandit M, Tudu VK, Chakravarty MK, Narayan SC, Rana M, Moharana D. Impact of water deficit stress in maize: Phenology and yield components. Sci Rep. 2020;10(1):2944. 32076012 10.1038/s41598-020-59689-7 PMC7031221

[5] Kim KH, Lee BM. Effects of Climate Change and Drought Tolerance on Maize Growth. Plants. 2023;12(20):3548. 37896012 10.3390/plants12203548 PMC10610049

[6] Nam HH, Seo MC, Cho HS, Lee YH, Seo YJ. Growth and yield responses of corn (*Zea mays* L.) as affected by growth period and irrigation intensity. Korean J Soil Sci Fertilizer. 2017;50(6):674–83.

[7] Effendi R, Priyanto SB, Aqil M, Azrai M. Drought adaptation level of maize genotypes based on leaf rolling, temperature, relative moisture content, and grain yield parameters. IOP Conference Series: Earth and Environmental Science. 2019;270( 1):012016. IOP Publishing.

[8] Badr A, El-Shazly HH. Climate Change and Biodiversity Loss: Interconnected Challenges and Priority Measures. CATRINA. 2024;29(1):69–78.

[9] Ali H, Ghori Z, Sheikh S, Gul A. Effects of gamma radiation on crop production. Crop production and global environmental issues. 2015;27–78. 10.1007/978-3-319-23162-4_2.

[10] Kovacs E, Keresztes A. Effect of gamma and UV-B/C radiation on plant cell. Micron. 2002;33:199–210. 11567888 10.1016/s0968-4328(01)00012-9

[11] Ashraf M, Cheema AA, Rashid M, Qamar Z. Effect of gamma rays on M1 generation in Basmati rice. Pak J Bot. 2003;35(5):791–5.

[12] Ashraf M. Biotechnological approach of improving plant salt tolerance using antioxidants as markers. Biotechnol Adv. 2009;27:84–93. 18950697 10.1016/j.biotechadv.2008.09.003

[13] El-Beltagi HS, Ahmed OK, El-Desouky W. Effect of low doses γ-irradiation on oxidative stress and secondary metabolites production of rosemary (*Rosmarinus officinalis* L.) callus culture. Radiat Phys Chem. 2011;80:968–976.

[14] Yadav A, Singh B, Singh SD. Impact of gamma irradiation on growth, yield and physiological attributes of maize. Indian J Exp Biol. 2019;57:116–22.

[15] Grant WF. Higher plant assays for the detection of chromosomal aberrations and gene mutations - A brief historical background on their use for screening and monitoring environmental chemicals. Mutat Res. 1999;426:107–12. 10350580 10.1016/s0027-5107(99)00050-0

[16] Auger DL, Sheridan WF. Plant chromosomal deletions, insertions and rearrangements. In: "Plant Cytogenetics: Genome Structure and Chromosome Function", Bass, H.W. and Birchler, J.A. (Ed.), 2011; pp.3–36. Plant Genetics and Genomics: Crops and Models Series Vol. 4. Springer Verlag.

[17] El-Azab EM, Soliman M, Soliman E, Badr A. Cytogenetic impact of gamma irradiation and its effects on growth and yield of three soybean cultivars. Egypt J Bot. 2018;58(3):411–22.

[18] Tan EH, Henry IM, Ravi M, Bradnam KR, Mandakova T, Marimuthu MP, Chan SW. Catastrophic chromosomal restructuring during genome elimination in plants. Elife. 2015;4:e06516. 25977984 10.7554/eLife.06516 PMC4461816

[19] Iqbal JA, Shinwari ZK, Rabbani MA, Khan SA. Genetic variability assessment of maize (*Zea mays* L.) germplasm based on total seed storage proteins banding pattern using SDS-PAGE. Eur. Acad. Res. 2014;2(2),2144–2160.

[20] Ali AH, Ahmed SA, Abdu-Allah GA, El-Sagheer SM. Effect of Some Herbicides on the Total Seed Proteins Patterns of Maize and *Sorghum* Using Sodium Dodecyl Sulphate Polyacrylamide Gel Electrophoresis (SDS–PAGE). Assiut J Agric Sci. 2023;54(2):190–201.

[21] Hasan AK, AL-Musawi BH. Biochemical Study of maize (*Zea mays* L.) genotypes through total seed protein by SDS-PAGE. J Kerbala Agric Sci. 2023;10(4):119–128.

[22] Mohammadkhani N, Heidari R. Effects of drought stress on soluble proteins in two maize varieties. Turk J Biol. 2008;32(1):23–30.

[23] Ko DK, Brandizzi F. Network-based approaches for understanding gene regulation and function in plants. Plant J. 2020;104(2):302–17. 32717108 10.1111/tpj.14940 PMC8922287

[24] Cheng C, An L, Li F, Ahmad W, Aslam M, Ul Haq MZ, Yan Y, Ahmad RM. Wide-range portrayal of AP2/ERF transcription factor family in maize (*Zea mays* L.) development and stress responses. Genes. 2023;14(1):194. 10.3390/genes14010194 PMC9859492 36672935

[25] Ashrafi M, Moqadam MRA, Moradi P, Mohsenifard E, Shekari F. Evaluation and validation of housekeeping genes in two contrast species of thyme plant to drought stress using real-time PCR. Plant Physiol Biochem. 2018;132:54–60. 30172853 10.1016/j.plaphy.2018.08.007

[26] Hassan AM, Ahmed MF, Rashed MA. Performance and transcriptomic analysis of Sorghum bicolor responding to drought stress. SABRAO J Breed Genet. 2022;54(4):814–25.

[27] Martins PK, Mafra V, de Souza WR, Ribeiro AP, Vinecky F, Basso MF, Molinari HBC. Selection of reliable reference genes for RT-qPCR analysis during developmental stages and abiotic stress in *Setaria viridis*. Sci Rep. 2016;6(1):28348. 10.1038/srep28348 PMC4913262 27321675

[28] Darlington CD, La Cour LF. The Handling of Chromosomes. 6th ed. London: George Allen and Unwin Ltd.; 1976.

[29] ISTA. International rules for seed testing: International Seed Testing Association (ISTA). Seed Sci Technol (Supplement). 1999;27.

[30] Livak KJ, Schmittgen TD. Analysis of relative gene expression data using real-time quantitative PCR and the 2− ΔΔCT method. Methods. 2001;25(4):402–8. 11846609 10.1006/meth.2001.1262

[31] Zamir MSI, Ahmad AH, Javeed HMR, Latif T. Growth and yield behavior of two maize hybrids (*Zea mays* L.) towards different plant spacing. Cercetări Agronom Moldova. 2011;XLIV(2):146.

[32] Laemmli UK. Cleavage of structural proteins during the assembly of the head of bacteriophage T4. Nature. 1970;227:680–6. 5432063 10.1038/227680a0

[33] Studier FW. Analysis of bacteriophage T7 early RNAs and proteins on slab gels. J Mol Biol. 1973;79:237–48. 4760132 10.1016/0022-2836(73)90003-x

[34] Hamideldin N, Eliwa NE. Gamma radiation and sodium azide influence on physiological aspects of maize under drought condition. Basic Rese J Agric Scie Review. 2015;4(1):5–13.

[35] Ahuja S, Kumar M, Kumar P, Gupta VK, Singhal RK, Yadav A, Singh B. Metabolic and biochemical changes caused by gamma irradiation in plants. Journal of Radio-analytical and Nuclear Chemistry. 2014;300:199–212.

[36] Azad ZR, Ahmad Khah M, Alam Q. Gamma irradiation induced chromosome aberrations in meiotic cells of bread wheat (*Triticum aestivum* L.). Int. J. Plant Soil Sci. 2022;26:81–89.

[37] Viccini LF, De Carvalho CR. Analysis of gamma radiation-induced chromosome variations in maize (*Zea mays* L.). Caryologia. 2001;54(4):319–327.

[38] Mahmoud AA, Galal AR, Ismail E, Ali SM, Ali AM, Nehal GA, Asaad NA, Abuzaid SA, Abdelraouf MA, Haitham MA. Influence of NaCl and PEG6000 Stresses on the Mitotic Chromosomal Biology of Bread Wheat Genotypes. Asian J Res Rev Agric. 2020;29–34.

[39] Brunner H. Radiation induced mutations for plant selection. Appl Radiat Isot. 1995;46(6–7):589–94.

[40] Yadav V. Effect of gamma radiation on various growth parameters and biomass of *Canscora decurrens Dalz*. Int J Herbal Med. 2016;4(5):109–15.

[41] Kazakova E, Gorbatova I, Khanova A, Shesterikova E, Pishenin I, Prazyan A, Volkova P. Radiation Hormesis in Barley Manifests as Changes in Growth Dynamics Coordinated with the Expression of PM19L-like, CML31-like, and AOS2-like. Int J Molecular Sci. 2024;25(2):974. 10.3390/ijms25020974 PMC10815718 38256048

[42] Jan S, Parween T, Siddiqi TO, Mahmooduzzafar. Effect of gamma radiation on morphological, biochemical, and physiological aspects of plants and plant products. Environ Rev. 2012;20(1):17–39.

[43] Liu S, Zenda T, Dong A, Yang Y, Liu X, Wang Y. Comparative proteomic and morphophysiological analyses of maize wildtype Vp and mutant vp16 germinating seed responses to PEG-induced drought stress. Int j Mol Sci. 2019;20(22):5586. 31717328 10.3390/ijms20225586 PMC6888951

[44] Badr A, ElShazly HH, Tarawneh RA, Börner A. Screening for drought tolerance in maize (*Zea mays* L.) germplasm using germination and seedling traits under simulated drought conditions. Plants. 2020;9(5):565. 32365550 10.3390/plants9050565 PMC7284379

[45] Falahati A, Kazemitabar SK, Bahrami AR, Lahouti M, Rahimi MF. The study of gamma irradiation effects on drought tolerance in rice (*Oryza sativa* L.). Indian J Crop Sci. 2007;2(1):155–8.

[46] Afram Y, Amenorpe G, Bediako EA, Darkwa AA, Amegbor IK. Assessing the sensitivity of maize genotypes to gamma radiation for germination and physiological characteristics. J Environ Radioact. 2024;271:107318. 10.1016/j.jenvrad.2023.107318 39492171

[47] Koutoua A, N’guessan K, Dogniméton S, Soumaïla O, Blaise KAEE, Nadia KA, Justin KY. Impact of drought on the foliar physiology of maize plants irradiated with gamma radiation. Int J Plant Physiol Bioch. 2021;13(2):30–7.

[48] Kiani D, Borzouei A, Ramezanpour S, Soltanloo H, Saadati S. Application of gamma irradiation on morphological, biochemical, and molecular aspects of wheat (*Triticum aestivum* L.) under different seed moisture contents. Sci Rep. 2022;12(1):11082. 35773375 10.1038/s41598-022-14949-6 PMC9246975

[49] Mizoi J, Shinozaki K, Yamaguchi-Shinozaki K. AP2/ERF family transcription factors in plant abiotic stress responses. Biochimica et Biophysica Acta (BBA)-Gene Regulatory Mechanisms. 2012;1819(2):86–96. 10.1016/j.bbagrm.2011.08.004 21867785

[50] Zhang Q, Liu H, Wu X, Wang W. Identification of drought tolerant mechanisms in a drought-tolerant maize mutant based on physiological, biochemical and transcriptomic analyses. BMC Plant Biol. 2020;20:1–14. 32620139 10.1186/s12870-020-02526-w PMC7350183

[51] Ying Wu, Li X, Zhang J, Zhao H, Tan S, Wanhao Xu, Pan J, Yang F, Pi E. ERF subfamily transcription factors and their function in plant responses to abiotic stresses. Front Plant Sci. 2022;13:1042084. 36531407 10.3389/fpls.2022.1042084 PMC9748296

[52] Gu Q, Kang J, Gao S, Zhao Y, Yi H, Zha X. Eukaryotic Translation Elongation Factor *OsEF1A* Positively Regulates Drought Tolerance and Yield in Rice. Plants. 2023;12(14):2593. 37514208 10.3390/plants12142593 PMC10383209

[53] Carneiro NP, Hughes PA, Larkins BA. The eEFlA gene family is differentially expressed in maize endosperm. Plant Mol Biol. 1999;41:801–14. 10737145 10.1023/a:1006391207980

[54] Nakashima K, Yamaguchi-Shinozaki K, Shinozaki K. The transcriptional regulatory network in the drought response and its crosstalk in abiotic stress responses including drought, cold, and heat. Front Plant Sci. 2014;5:170. 24904597 10.3389/fpls.2014.00170 PMC4032904

[55] Zafar SA, Maqbool A, Naeem M. Mutagenic effectiveness of gamma rays in inducing polygenic variability in maize (*Zea mays* L.). J Agric Basic Sci. 2020;5(1):14–23.

[56] Francois Konan YK, Koutoua A, Dogniméton S, Kissomanbien KS, Blaise KA, Justin KY. Effect of Gamma Irradiation of Seeds on the Development and Productivity of Three Maize Varieties (*Zea mays* L.). Asian Res J Agric. 2021;14(4):89–99.

[57] Khan M, Zulqarnain Khan M, Muhammad P, Abubakkar Bilal M, Usman M, Adil M. Impact of Gamma Rays Induced Mutations on Morphological and Yield attributing Characters in Maize (*Zea Mays* L). Biosci Res. 2024;21(3):595–610.

[58] Shailaja DS, Lohithaswa HC, Sowmya MS, Mallikarjuna MG, Banakara S, Likhithashree TR, Kirankumar R, Basanagouda G, Patne N, Vivek BS. Identification of drought tolerant inbred lines and assessment of combining ability in maize (*Zea mays* L. Plant Breeding. 2024;143(4):562–86.

[59] Katiyar P, Pandey N, Keshavkant S. Gamma radiation: A potential tool for abiotic stress mitigation and management of agroecosystem. Plant stress. 2022;5:100089.

[60] Romero-Galindo R, Hernández-Aguilar C, Dominguez-Pacheco A, Godina-Nava JJ, Tsonchev RI. Biophysical methods used to generate tolerance to drought stress in seeds and plants: a review. International Agrophysics. 2022;35(4):389–410.

[61] Adly M, El-Fiki A. Genetic diversity in *Triticum aestivum* L. induced by gamma irradiation and selection for drought stress by using PEG 6000. J Nuclear Technol Applied Sci. 2016;4(3):157–67.

[62] Bharat RA, Prathmesh SP, Sarsu F, Suprasanna P. Induced Mutagenesis using Gamma Rays: Biological Features and Applications in Crop Improvement. OBM Genetics. 2024;8(2):1–27.

[63] Rashad SE, Heiba SA, Emam MA, Osman SA, Eldemerdash IS. Effect of PEG induced drought stress on Genetic diversity using SDS-PAGE and ISSR markers for Egyptian barley varieties. Egypt J Chem. 2023;66(13):647–58.

[64] Yang L, Jiang T, Fountain JC, Scully BT, Lee RD, Kemerait RC, Chen S, Guo B. Protein profiles reveal diverse responsive signaling pathways in kernels of two maize inbred lines with contrasting drought sensitivity. Int J Mol Sci. 2014;15(10):18892–918. 25334062 10.3390/ijms151018892 PMC4227252

[65] Zhang C, Liu Y, Feng C, Wang Q, Shi H, Zhao D, Yu R, Su Z. Loss of PEG chain in routine SDS-PAGE analysis of PEG-maleimide modified protein. Electrophoresis. 2015;36(2):371–4. 25265901 10.1002/elps.201400373

[66] Batool A, Yue DX, Xiao YL, Li SS, Duan HX, Haq Z, Ahmed K, Zhao L, Zhu L, Xiong YC. Plant tolerance to drought stress: Complexity and mechanism across physiological, molecular and biochemical scales. Int J Applied Experimental Biol. 2024;3(2):159–75.

